# Items

**Published:** 1901-01

**Authors:** 


### ITEMS.

SCOTT & BOWNE have presented us with their New Twentieth Century Calendar and memorandum for 1901. It is one of the most convenient articles of desk furniture and is a daily reminder of the value of Scott's emulsion as a nutritive and reconstructive agent.

---

THE Columbia Calendar for 1901 is placed upon our desk promptly by the American Bicycle Company, dealers in Columbia and Hartford cycles. It is unique in many respects, the sentiment for each day being supplied by bicycle riders. Send 10 cents in stamps to the Company, at Hartford, Conn., and get one.

---

THE Unique Card Index System of accounts, case records, therapeutics, advances in surgery and allied sciences is issued by the Technique Publishing Co., of New York. Information concerning it will be supplied upon application to the Company.

---

THE Antikamnia Calendar for 1901 has come to hand. It is issued in four tablets, each sheet containing a calendar for three months and each is illustrated with a skeleton sketch, painted in water color by the late Dr. L. Crusius. These four are the last of the "sketches" left by Dr. Crusius at his death. They are remarkable specimens of an art that is unique—that of giving life-like facial expression to the human skull.

